# Tumour Evolution Driving Genome Instability, Immune Interactions, and Response to Radiotherapy

**DOI:** 10.1097/PPO.0000000000000777

**Published:** 2025-08-11

**Authors:** George Seed, France Truong, Rahma Riahi, Ben O’Leary

**Affiliations:** 1 The Institute of Cancer Research, London, UK; 2 Centre Hospitalo-Universitaire de Reims, Reims, France; 3 Université Grenoble Alpes, Grenoble, France; 4 The Royal Marsden Hospital, London, UK

**Keywords:** radiotherapy, immunotherapy, cancer evolution, genomic instability, immune evasion, immune surveillance

## Abstract

This review article explores the role of immuno-radiotherapy in the context of genome instability and tumour evolution. Genomic changes in tumours exist in a delicate balance with the immune system, offering evolutionary pathways to adapt and grow but risking provoking an immune response. Rapid developments across both immunotherapy and radiotherapy have raised questions about the potential benefits combination therapy, and how best to identify ideal treatment populations. Here we discuss foundational studies of genomic instability and tumour evolution, how these paradigms translate into immune surveillance and evasion, and subsequently go on to explore recent preclinical and clinical studies of both treatment modalities. Understanding how cancers evolve in the context of the immune system could provide a key insight in delivering better therapies that could overcome treatment resistance.

## Introduction

Radiotherapy is a key part of curative treatment for many tumour types, featuring in the management of around 40% of cancer patients, and is often used alongside other treatment modalities with curative intent ^1^. Despite this ubiquity, the mechanisms underlying resistance and sensitivity are still poorly understood. The anti-cancer effect of radiotherapy is thought to be mediated in large part by direct DNA damage, but recent work increasingly highlights an important role for immune activation in this process ^2–5^.

Tumours evolve from normal cells, in part through DNA changes which arise due to genome instability, and this capacity for random mutations to proliferate in cell populations is a key ‘enabling characteristic’ for tumours, fuelling the acquisition of events that drive other oncogenic hallmarks ^6^. This paradigm is detectable *via* DNA sequencing in several forms ranging from single nucleotide variants to large scale copy-number alterations and aneuploidy, which taken together can be used to infer the presence of historic genome instability, although it is more challenging to use this data to identify ongoing instability with confidence ^7,8^.

These ongoing genomic changes set up a dynamic equilibrium between the tumour and its associated microenvironment, where DNA damage and aberrations provoke an immune system response, requiring tumours to develop mechanisms to evade detection. With radiotherapy exerting dual effects through DNA damage and immune stimulation, this, therefore, presents an appealing rationale for combining immuno-stimulatory or -modulatory agents alongside radiotherapy, to damage DNA and stimulate an immune response to the tumour alongside a reduction in the ability of the tumour to evade surveillance.

In this review, we will consider recent evidence linking cancer evolution through genomic instability with immune evasion, and its applicability to radiotherapy resistance in the clinical setting.

## Genomic Instability Fuels Cancer Evolution

Tumour evolution is the result of individual cells accruing alterations and these, alongside environmental pressures, acting to shape tumour composition over time. Mutations that offer advantages are preferentially selected over time, fuelling expansion of the fittest individual sub-clonal cell populations. As sequencing technologies have developed over the last two decades, so has our knowledge of tumour clonality and heterogeneity, with studies demonstrating that highly diverse, rapidly changing tumours result in rapid disease progression and poor prognosis ^9–12^. The accrual of changes over time in somatic tumours has been revealed by longitudinal and multi-sample sequencing studies, and these are reflective of underlying evolutionary forces acting on the tumour ^13,14^. Genome instability is multi-faceted, not solely limited to DNA aberrations, with substantial and growing evidence for the importance of epigenetic effects, though we will not consider these here ^15^.

DNA is damaged by diverse mechanisms, both endogenous and exogenous, and these lesions are subsequently repaired by internal cellular machinery ^16^. This can be explored in aggregate via signature methods, which leverage the fact that different damage and repair mechanisms leave characteristic traces in the genome that can be used to infer underlying mutational processes ^17–21^ and potentially serve as clinical biomarkers ^22,23^.

There are limits to a cell’s ability to tolerate genomic changes, however, even in the case of tumours ^24^. There is evidence that tumours that exhibit a total loss of control over the genome and massive levels of copy number changes or small variants are associated with better disease outcomes in observational studies ^25–29^. This has been effectively demonstrated as a therapeutic vulnerability when targeting DNA repair processes such as homologous repair deficiency ^30–32^. Pronounced genomic instability may, therefore, confer a favourable prognosis in certain cancer types and treatment contexts.

## Rising Tensions with the Immune System

The immune system carries out a crucial role in monitoring tissues, recognising and destroying aberrant cells that could develop into tumours; demonstrated first experimentally by studies of mice lacking key immune components having a high susceptibility to multiple malignancies ^33–37^. These studies are complemented by numerous observations that immunocompromised patients present with tumours more frequently than the general population ^38,39^.

Damage to DNA has several knock-on effects which can stimulate the immune system, highlighting the tumour for destruction ^40^. Detection of abnormal nucleic acids in the cytosol is mediated by internal sensors, such as cyclic GMP–AMP synthase (cGAS), Toll-like receptor 9 (TLR9) and absent in melanoma 2 (AIM2) ^41,42^. Recognition by these pathways leads to inflammation, activation of the immune system and the attraction of CD8_+_ T-cells into the tumour ^43–46^.

As a complement to this, genome instability leads to the generation of aberrant proteins, which once presented by the major histocompatibility complex (MHC) can further act as a signal to the immune system, with these neo-antigens recognised by T-cells as non-self and leading to the cell that expresses them being destroyed ^47^. DNA repair-deficient tumours with high numbers of somatic variants are associated with a greater degree of immune infiltration, and infiltration of these cells into tumours is associated with better prognosis, although the precise mechanisms in any individual case are often unclear ^48^. Immune surveillance, therefore, effectively works as another selective pressure on tumours.

## Under the Radar: Mechanisms of Immune Evasion

Due to the immune system recognising and targeting pre-malignant and malignant tumours, only those able to avoid this predation are likely to survive, a process commonly termed ‘immune evasion’ or ‘immune escape’. The presence of immune cells infiltrating tumours, alongside markers of T-cell migration, has been shown to be a prognostic factor in numerous studies, with ‘immune cold’ tumours typically having a poor prognosis ^49,50^. Tumour aneuploidy – linked with overall genome instability – is associated with reduced expression of genes linked with cytotoxic T-cells and reductions in immune infiltration, providing clear evidence for an association between genome instability and evolved immune evasion ^51,52^.

A variety of mechanisms have been identified for this paradigm, acting at tumour-intrinsic and -extrinsic levels, which has important implications for tumour biology and treatment opportunities ^53,54^. At the local level, tumour cells can act through direct cell-cell communication, for example by modifying HLA presentation and increasing PD-L1 expression, rendering the offending cells less detectable by patrolling immune cells ^55–60^. Tumour cells that lose cGAS-STING signalling exhibit a reduction in their ability to signal to the immune system as a response to DNA damage ^61^. This has also been observed in the case of disruption to genes governing downstream processes, such as loss of a 9p locus containing type-1 interferon-related genes ^62,63^. Tumours can also act more indirectly, for example by suppressing the production of chemo-attractants such as CXCL9 and CXCL10, which thereby limits the migration of T-cells into the tumour microenvironment ^64,65^. Several studies have shown that the expression of pro-angiogenic factors such as VEGF acts as a barrier to immune migration by limiting the capacity of nearby endothelial cells to mediate T-cell transfer from the blood circulation ^66,67^. Nearby healthy cells, such as fibroblasts, can offer protection to tumours through remodelling of the extracellular matrix and production of chemo-repulsive cytokines such as CXCL12, and these behaviours can be induced by TGFβ expression by the tumour ^68–70^.

More distantly, tumours with increased WNT–β-catenin signalling have been demonstrated to reduce recruitment of dendritic cells (DCs), which are key for detection of antigens and priming T-cells ^71–73^. Systemic immune suppression has also been described, mediated by the release of cytokines that modify the production of immune cell populations in peripheral tissues, suppressing an antitumour response ^72,74–76^. As tumours evolve, these diverse mechanisms must be adapted to the unique circumstances of each microenvironment ^77^.

## Therapeutic Pressures Reshaping Genomic and Immune Landscapes

Both radiotherapy and immunotherapy have been shown to interact with the above paradigms in important ways, with potential therapeutic implications. The DNA-damaging effects of ionising radiation are well-characterised, both through direct reaction with DNA molecules but also *via* the generation of reactive oxygen species ^78,79^. This damage yields similar effects to genomic instability and provokes repair mechanisms in line with other sources of DNA damage ^80,81^. Further, sequencing studies have detected distinct signatures of radiation-induced mutations in post-radiotherapy tumours, suggesting that even in radioresistant tumours, DNA damage persists in the cell population and contributes to an overall burden of genome instability ^4,82^.

In addition to the direct mechanisms, indirect effects of radiotherapy on the immune system have also been identified ^83–85^. The stress of ionizing radiation can effectively spotlight tumour cells for immune surveillance, both by increasing tumour antigen production and availability but also through proinflammatory signalling and remodelling of the local microenvironment and vascular system ^86–89^. These factors also help to explain the abscopal effect, tumour regression outside of the irradiated region, which is driven by immune cells primed by the initial wave of ionising radiation that go on to attack non-irradiated lesions, though rarely observed in the clinic ^90,91^. However, conflicting data also shows that radiotherapy can also result in immune-suppressing effects, which could limit an immune response ^92,93^.

These data suggest that radiotherapy shares a key overlap with immunotherapy in modulating tumour-immune interactions. All tumours employ methods of immune escape to varying degrees, and the goal of immunotherapy is to tip the balance against the tumour in favour of the host immune system ^94,95^. Many different therapeutic approaches have been taken to immunotherapy with variable outcomes, supporting an approach that more closely considers the specific immune evasion mechanisms. Some tumours interact with specific immune checkpoints (i.e. PDL-1 and CTLA-4) directly to suppress T-cells and targeting these mechanisms has shown marked responses to therapy and improved prognoses in several tumour types; indeed, these are undoubtedly the most clinically mature class of immunotherapies available to date ^96–98^. The relationship between responses to these agents and immune escape is unclear, though the commonly used biomarkers such as Tumor Proportion Score and Combined Positive Score both use PD-L1 staining on cells as a proxy measure for immune engagement. Tumours with high genome instability (increased mutation load through MMR DNA repair deficiency) show increased responses to these checkpoint inhibitors, presumably due to a higher existing tension with the immune system ^99,100^. Other therapies aim to boost a systemic immune response, such as through the Interleukin-2 (IL-2) cytokine, which promotes the activation and growth of T- and NK-cells, but it is unclear how best to target these based on specific mechanisms of immune escape ^101^.

Approaches have also been developed that aim to boost the activity of specific cellular populations. Sipuleucel-T is an early example of this, approved in 2010 by the FDA for prostate cancer, and involves the extraction and priming of dendritic cells using an immune cell growth factor fused to a prostate cell antigen ^102–104^. Recent advances in genome editing have led to CAR-T cell therapy, in which T-cells are engineered to recognise an individual tumour antigen, which is approved for use in haematological cancers and, in some cases, is capable of resulting in complete remission ^105–108^. Targeting specific immune subpopulations in this way could provide an approach to addressing specific immune escape mechanisms. As immunotherapy approaches become more complex, there is increasing urgency to identify ways of targeting them most effectively.

## Attacking from Both Directions: Combining Radiotherapy with Immunotherapy

Radiotherapy (RT) is delivered for purposes of local control but, as mentioned above, also acts as a trigger of systemic antitumor immune response, offering an appealing rationale to combine with immunotherapies ^3,109–111^. There is mounting evidence that the effects of these approaches can be synergistic, and emerging studies have explored this combination for patients with advanced tumours ^112^.

Exposure to radiation induces changes to cell surface expression of MHC class I molecules resulting in antigen presentation and immune recognition of irradiated cells, and was shown to boost the efficacy of adoptive CTL immunotherapy *in vivo* when tested on a murine colon adenocarcinoma ^113^. Supporting this paradigm, a phase II clinical trial of Sipuleucel-T with a CTLA-4 inhibitor (Ipilimumab) found that prior radiotherapy was associated with an improved prognosis, along with lower frequencies of CTLA-4-positive T-cells in peripheral blood ^114^. Similarly, a phase 1/1b trial of hypofractionated stereotactic body radiation therapy (SBRT) with single-dose anti-PD-L1 immunotherapy neoadjuvantly in head and neck tumours found clear signs of immunomodulation and antigen presentation, alongside the highest response rate yet observed in this tumour type ^115^.

The PACIFIC phase III trial compared durvalumab with placebo in patients with unresectable, stage III non-small-cell lung cancer with no disease progression after curative-intent chemoradiotherapy. Overall survival and PFS benefit were found in those patients who received durvalumab as consolidation, adjuvant treatment ^116^. However, these results conflicted with the subsequent PACIFIC-2 phase-III trial, which evaluated concurrent immunotherapy alongside chemoradiotherapy that found no survival advantage compared to chemoradiotherapy alone, suggesting that treatment order could be of critical importance in this setting ^117^. Further, the JAVELIN Head and Neck 100 trial found similar results, with concurrent immuno-radiotherapy offering no advantage compared to radiotherapy alone in head and neck cancer ^118,119^.

These, and other conflicting studies, may be partly explained by differences in treatment regimens; for example, Telarovic *et* al found that by delaying draining lymph nodes ionizing radiation (DLN-IR) they reduced detrimental effects while retaining the beneficial effect of subsequent DLN-IR on metastatic tumour cell killing ^120^. Shen *et* al recently showed that radiotherapy had a profound effect on T-cell migration from the DLN and that this was a key mediator of tumour control ^121^. Sparing lymph nodes may, therefore, also be key to developing durable responses to immunotherapy in line with studies exploring the abscopal effect ^111^. Intriguingly, Chen *et al* explored low-dose (1 Gy) intestinal radiation alongside immune checkpoint inhibition and found improved survival in patient data alongside gut emigration of PD-L1-expressing immune cells in mouse models ^122^.

The mixed results from these data provide a clear argument for better understanding the relationship between radiotherapy and the immune system.

## In Summary

Radiotherapy and immunotherapy, despite emerging from distinct developmental trajectories, have become cornerstones of cancer treatment, each capable of achieving durable tumor control in appropriate clinical settings. Both modalities are intrinsically linked to genomic instability—radiotherapy acts to augment existing instability, while immunotherapy leverages the immune system's ability to recognize tumors, partly through signals associated with accumulating genomic damage. Although combining these approaches remains mechanistically promising, clinical implementation has proven more challenging than initial preclinical data suggested. Failure of several high-profile trials has prompted critical reassessment of patient selection and trial design strategies. Recent data, however, illuminates potential pathways to optimize future studies and improve response rates by harnessing the synergistic mechanisms of both therapies. Understanding the co-evolution of genomic instability and immune evasion could provide crucial insights for effectively targeting cancers with radiotherapy and immunotherapy, ultimately supporting next-generation precision medicine approaches.

## References

[1] Mee T, Kirkby NF, Defourny NN, Kirkby KJ, Burnet NG (2023). The use of radiotherapy, surgery and chemotherapy in the curative treatment of cancer: results from the FORTY (Favourable Outcomes from RadioTherapY) project. British Journal of Radiology.

[2] Wennerberg E, Vanpouille-Box C, Bornstein S, Yamazaki T, Demaria S, Galluzzi L (2017). Immune recognition of irradiated cancer cells. Immunological Reviews.

[3] Balázs K, Kis E, Badie C (2019). Radiotherapy-Induced Changes in the Systemic Immune and Inflammation Parameters of Head and Neck Cancer Patients. Cancers (Basel).

[4] Behjati S, Gundem G, Wedge DC (2016). Mutational signatures of ionizing radiation in second malignancies. Nature Communications.

[5] Frankenberg-Schwager M (1990). Induction, repair and biological relevance of radiation-induced DNA lesions in eukaryotic cells. Radiation and Environmental Biophysics.

[6] Hanahan D, Weinberg RA (2011). Hallmarks of cancer: the next generation. Cell.

[7] Hegan DC, Narayanan L, Jirik FR, Edelmann W, Liskay RM, Glazer PM (2006). Differing patterns of genetic instability in mice deficient in the mismatch repair genes Pms2, Mlh1, Msh2, Msh3 and Msh6. Carcinogenesis.

[8] Sansregret L, Swanton C (2017). The Role of Aneuploidy in Cancer Evolution. Cold Spring Harb Perspect Med.

[9] Jamal-Hanjani M, Wilson GA, McGranahan N (2017). Tracking the Evolution of Non-Small-Cell Lung Cancer. N Engl J Med.

[10] Frankell AM, Dietzen M, Al Bakir M (2023). The evolution of lung cancer and impact of subclonal selection in TRACERx. Nature.

[11] Almendro V, Cheng YK, Randles A (2014). Inference of tumor evolution during chemotherapy by computational modeling and in situ analysis of genetic and phenotypic cellular diversity. Cell Rep.

[12] Merlo LM, Shah NA, Li X (2010). A comprehensive survey of clonal diversity measures in Barrett's esophagus as biomarkers of progression to esophageal adenocarcinoma. Cancer Prev Res (Phila).

[13] Jamal-Hanjani M, Hackshaw A, Ngai Y (2014). Tracking genomic cancer evolution for precision medicine: the lung TRACERx study. PLoS Biol.

[14] Al Bakir M, Huebner A, Martínez-Ruiz C (2023). The evolution of non-small cell lung cancer metastases in TRACERx. Nature.

[15] Heide T, Househam J, Cresswell GD (2022). The co-evolution of the genome and epigenome in colorectal cancer. Nature.

[16] Hoeijmakers JH (2009). DNA damage, aging, and cancer. N Engl J Med.

[17] Alexandrov LB, Nik-Zainal S, Wedge DC (2013). Signatures of mutational processes in human cancer. Nature.

[18] Nik-Zainal S, Alexandrov Ludmil B, Wedge David C (2012). Mutational Processes Molding the Genomes of 21 Breast Cancers. Cell.

[19] Steele CD, Abbasi A, Islam SMA (2022). Signatures of copy number alterations in human cancer. Nature.

[20] Macintyre G, Goranova TE, De Silva D (2018). Copy number signatures and mutational processes in ovarian carcinoma. Nat Genet.

[21] Drews RM, Hernando B, Tarabichi M (2022). A pan-cancer compendium of chromosomal instability. Nature.

[22] Essers PBM, van der Heijden M, Vossen D (2020). Ovarian cancer-derived copy number alterations signatures are prognostic in chemoradiotherapy-treated head and neck squamous cell carcinoma. Int J Cancer.

[23] Pham TV, Boichard A, Goodman A (2020). Role of ultraviolet mutational signature versus tumor mutation burden in predicting response to immunotherapy. Molecular Oncology.

[24] Andor N, Maley CC, Ji HP (2017). Genomic Instability in Cancer: Teetering on the Limit of Tolerance. Cancer Research.

[25] Ohashi A, Ohori M, Iwai K (2015). Aneuploidy generates proteotoxic stress and DNA damage concurrently with p53-mediated post-mitotic apoptosis in SAC-impaired cells. Nat Commun.

[26] Santaguida S, Richardson A, Iyer DR (2017). Chromosome Mis-segregation Generates Cell-Cycle-Arrested Cells with Complex Karyotypes that Are Eliminated by the Immune System. Dev Cell.

[27] Krivega M, Stiefel CM, Karbassi S (2021). Genotoxic stress in constitutive trisomies induces autophagy and the innate immune response via the cGAS-STING pathway. Commun Biol.

[28] Birkbak NJ, Eklund AC, Li Q (2011). Paradoxical relationship between chromosomal instability and survival outcome in cancer. Cancer Res.

[29] Jamal-Hanjani M, A'Hern R, Birkbak NJ (2015). Extreme chromosomal instability forecasts improved outcome in ER-negative breast cancer: a prospective validation cohort study from the TACT trial. Ann Oncol.

[30] Mateo J, Carreira S, Sandhu S (2015). DNA-Repair Defects and Olaparib in Metastatic Prostate Cancer. New England Journal of Medicine.

[31] Kaufman B, Shapira-Frommer R, Schmutzler RK (2015). Olaparib monotherapy in patients with advanced cancer and a germline BRCA1/2 mutation. J Clin Oncol.

[32] Farmer H, McCabe N, Lord CJ (2005). Targeting the DNA repair defect in BRCA mutant cells as a therapeutic strategy. Nature.

[33] Dunn GP, Bruce AT, Ikeda H, Old LJ, Schreiber RD (2002). Cancer immunoediting: from immunosurveillance to tumor escape. Nature Immunology.

[34] Girardi M, Oppenheim DE, Steele CR (2001). Regulation of cutaneous malignancy by gammadelta T cells. Science.

[35] Shankaran V, Ikeda H, Bruce AT (2001). IFNgamma and lymphocytes prevent primary tumour development and shape tumour immunogenicity. Nature.

[36] Smyth MJ, Thia KY, Street SE, MacGregor D, Godfrey DI, Trapani JA (2000). Perforin-mediated cytotoxicity is critical for surveillance of spontaneous lymphoma. J Exp Med.

[37] Street SE, Trapani JA, MacGregor D, Smyth MJ (2002). Suppression of lymphoma and epithelial malignancies effected by interferon gamma. J Exp Med.

[38] Gatti RA, Good RA (1971). Occurrence of malignancy in immunodeficiency diseases. A literature review. Cancer.

[39] Pham SM, Kormos RL, Landreneau RJ (1995). Solid tumors after heart transplantation: lethality of lung cancer. Ann Thorac Surg.

[40] Nastasi C, Mannarino L, D’Incalci M (2020). DNA Damage Response and Immune Defense. International Journal of Molecular Sciences.

[41] Motwani M, Pesiridis S, Fitzgerald KA (2019). DNA sensing by the cGAS–STING pathway in health and disease. Nature Reviews Genetics.

[42] Konno H, Yamauchi S, Berglund A, Putney RM, Mulé JJ, Barber GN (2018). Suppression of STING signaling through epigenetic silencing and missense mutation impedes DNA damage mediated cytokine production. Oncogene.

[43] Luecke S, Holleufer A, Christensen MH (2017). cGAS is activated by DNA in a length-dependent manner. EMBO Rep.

[44] Parkes EE, Walker SM, Taggart LE (2017). Activation of STING-Dependent Innate Immune Signaling By S-Phase-Specific DNA Damage in Breast Cancer. J Natl Cancer Inst.

[45] Kaneta A, Nakajima S, Okayama H (2022). Role of the cGAS-STING pathway in regulating the tumor-immune microenvironment in dMMR/MSI colorectal cancer. Cancer Immunol Immunother.

[46] Chen M, Yu S, van der Sluis T (2024). cGAS-STING pathway expression correlates with genomic instability and immune cell infiltration in breast cancer. npj Breast Cancer.

[47] Boissière-Michot F, Lazennec G, Frugier H (2014). Characterization of an adaptive immune response in microsatellite-instable colorectal cancer. Oncoimmunology.

[48] Giannakis M, Mu XJ, Shukla SA (2016). Genomic Correlates of Immune-Cell Infiltrates in Colorectal Carcinoma. Cell Rep.

[49] Pagès F, Berger A, Camus M (2005). Effector memory T cells, early metastasis, and survival in colorectal cancer. N Engl J Med.

[50] Reissfelder C, Stamova S, Gossmann C (2015). Tumor-specific cytotoxic T lymphocyte activity determines colorectal cancer patient prognosis. J Clin Invest.

[51] Davoli T, Uno H, Wooten EC, Elledge SJ (2017). Tumor aneuploidy correlates with markers of immune evasion and with reduced response to immunotherapy. Science.

[52] William WN, Zhao X, Bianchi JJ (2021). Immune evasion in HPV(-) head and neck precancer-cancer transition is driven by an aneuploid switch involving chromosome 9p loss. Proc Natl Acad Sci U S A.

[53] Angelova M, Mlecnik B, Vasaturo A (2018). Evolution of Metastases in Space and Time under Immune Selection. Cell.

[54] Roerden M, Spranger S (2025). Cancer immune evasion, immunoediting and intratumour heterogeneity. Nature Reviews Immunology.

[55] McGranahan N, Rosenthal R, Hiley CT (2017). Allele-Specific HLA Loss and Immune Escape in Lung Cancer Evolution. Cell.

[56] Nakanishi J, Wada Y, Matsumoto K, Azuma M, Kikuchi K, Ueda S (2007). Overexpression of B7-H1 (PD-L1) significantly associates with tumor grade and postoperative prognosis in human urothelial cancers. Cancer Immunol Immunother.

[57] Katsuya Y, Fujita Y, Horinouchi H, Ohe Y, Watanabe S, Tsuta K (2015). Immunohistochemical status of PD-L1 in thymoma and thymic carcinoma. Lung Cancer.

[58] Demanet C, Mulder A, Deneys V (2004). Down-regulation of HLA-A and HLA-Bw6, but not HLA-Bw4, allospecificities in leukemic cells: an escape mechanism from CTL and NK attack?. Blood.

[59] Alspach E, Lussier DM, Miceli AP (2019). MHC-II neoantigens shape tumour immunity and response to immunotherapy. Nature.

[60] Strand S, Hofmann WJ, Hug H (1996). Lymphocyte apoptosis induced by CD95 (APO-1/Fas) ligand-expressing tumor cells--a mechanism of immune evasion?. Nat Med.

[61] Guan Lu (2021).

[62] Ganguli P, Basanta CC, Acha-Sagredo A (2025). Context-dependent effects of CDKN2A and other 9p21 gene losses during the evolution of esophageal cancer. Nature Cancer.

[63] Zhao X, Liu B, William WN (2024). Interferon-ε loss is elusive 9p21 link to immune-cold tumors, resistant to immune-checkpoint therapy and endogenous CXCL9/10 induction. J Thorac Oncol.

[64] Weenink B, Draaisma K, Ooi HZ (2019). Low-grade glioma harbors few CD8 T cells, which is accompanied by decreased expression of chemo-attractants, not immunogenic antigens. Sci Rep.

[65] Harlin H, Meng Y, Peterson AC (2009). Chemokine expression in melanoma metastases associated with CD8_+_ T-cell recruitment. Cancer Res.

[66] Huang H, Langenkamp E, Georganaki M (2015). VEGF suppresses T-lymphocyte infiltration in the tumor microenvironment through inhibition of NF-κB-induced endothelial activation. Faseb j.

[67] Melder RJ, Koenig GC, Witwer BP, Safabakhsh N, Munn LL, Jain RK (1996). During angiogenesis, vascular endothelial growth factor and basic fibroblast growth factor regulate natural killer cell adhesion to tumor endothelium. Nat Med.

[68] Salmon H, Franciszkiewicz K, Damotte D (2012). Matrix architecture defines the preferential localization and migration of T cells into the stroma of human lung tumors. J Clin Invest.

[69] Risom T, Glass DR, Averbukh I (2022). Transition to invasive breast cancer is associated with progressive changes in the structure and composition of tumor stroma. Cell.

[70] Costa A, Kieffer Y, Scholer-Dahirel A (2018). Fibroblast Heterogeneity and Immunosuppressive Environment in Human Breast Cancer. Cancer Cell.

[71] Spranger S, Dai D, Horton B, Gajewski TF (2017). Tumor-Residing Batf3 Dendritic Cells Are Required for Effector T Cell Trafficking and Adoptive T Cell Therapy. Cancer Cell.

[72] Meyer MA, Baer JM, Knolhoff BL (2018). Breast and pancreatic cancer interrupt IRF8-dependent dendritic cell development to overcome immune surveillance. Nat Commun.

[73] de Galarreta Ruiz, Bresnahan E, Molina-Sánchez P (2019). β-Catenin Activation Promotes Immune Escape and Resistance to Anti-PD-1 Therapy in Hepatocellular Carcinoma. Cancer Discov.

[74] Wang L, Miyahira AK, Simons DL (2017). IL6 Signaling in Peripheral Blood T Cells Predicts Clinical Outcome in Breast Cancer. Cancer Res.

[75] Failli A, Legitimo A, Orsini G, Romanini A, Consolini R (2013). Numerical defect of circulating dendritic cell subsets and defective dendritic cell generation from monocytes of patients with advanced melanoma. Cancer Lett.

[76] Wang L, Simons DL, Lu X (2019). Connecting blood and intratumoral T(reg) cell activity in predicting future relapse in breast cancer. Nat Immunol.

[77] Chu X, Li X, Zhang Y (2024). Integrative single-cell analysis of human colorectal cancer reveals patient stratification with distinct immune evasion mechanisms. Nature Cancer.

[78] Kim W, Lee S, Seo D (2019). Cellular Stress Responses in Radiotherapy. Cells.

[79] Ross GM (1999). Induction of cell death by radiotherapy. Endocr Relat Cancer.

[80] Rübe CE, Grudzenski S, Kühne M (2008). DNA double-strand break repair of blood lymphocytes and normal tissues analysed in a preclinical mouse model: implications for radiosensitivity testing. Clin Cancer Res.

[81] Horn S, Barnard S, Rothkamm K (2011). Gamma-H2AX-based dose estimation for whole and partial body radiation exposure. PLoS One.

[82] Kocakavuk E, Anderson KJ, Varn FS (2021). Radiotherapy is associated with a deletion signature that contributes to poor outcomes in patients with cancer. Nat Genet.

[83] Lee Y, Auh SL, Wang Y (2009). Therapeutic effects of ablative radiation on local tumor require CD8_+_ T cells: changing strategies for cancer treatment. Blood.

[84] Schaue D, Ratikan JA, Iwamoto KS, McBride WH (2012). Maximizing tumor immunity with fractionated radiation. Int J Radiat Oncol Biol Phys.

[85] Gupta A, Probst HC, Vuong V (2012). Radiotherapy promotes tumor-specific effector CD8_+_ T cells via dendritic cell activation. J Immunol.

[86] Lin W, Xu Y, Chen X (2020). Radiation-induced small extracellular vesicles as "carriages" promote tumor antigen release and trigger antitumor immunity. Theranostics.

[87] Zhou Z, Zhao J, Hu K (2021). Single High-Dose Radiation Enhances Dendritic Cell Homing and T Cell Priming by Promoting Reactive Oxygen Species-Induced Cytoskeletal Reorganization. Int J Radiat Oncol Biol Phys.

[88] Lhuillier C, Rudqvist NP, Elemento O, Formenti SC, Demaria S (2019). Radiation therapy and anti-tumor immunity: exposing immunogenic mutations to the immune system. Genome Med.

[89] Matsumura S, Wang B, Kawashima N (2008). Radiation-induced CXCL16 release by breast cancer cells attracts effector T cells. J Immunol.

[90] Buchwald ZS, Nasti TH, Lee J (2020). Tumor-draining lymph node is important for a robust abscopal effect stimulated by radiotherapy. J Immunother Cancer.

[91] Rodríguez-Ruiz ME, Vanpouille-Box C, Melero I, Formenti SC, Demaria S (2018). Immunological Mechanisms Responsible for Radiation-Induced Abscopal Effect. Trends Immunol.

[92] Lin L, Kane N, Kobayashi N (2021). High-dose per Fraction Radiotherapy Induces Both Antitumor Immunity and Immunosuppressive Responses in Prostate Tumors. Clin Cancer Res.

[93] Tsai CS, Chen FH, Wang CC (2007). Macrophages from irradiated tumors express higher levels of iNOS, arginase-I and COX-2, and promote tumor growth. Int J Radiat Oncol Biol Phys.

[94] Kamrani A, Hosseinzadeh R, Shomali N (2023). New immunotherapeutic approaches for cancer treatment. Pathology - Research and Practice.

[95] Ling SP, Ming LC, Dhaliwal JS (2022). Role of Immunotherapy in the Treatment of Cancer: A Systematic Review. Cancers (Basel).

[96] Reck M, Rodríguez-Abreu D, Robinson AG (2016). Pembrolizumab versus Chemotherapy for PD-L1-Positive Non-Small-Cell Lung Cancer. N Engl J Med.

[97] Hodi FS, O'Day SJ, McDermott DF (2010). Improved survival with ipilimumab in patients with metastatic melanoma. N Engl J Med.

[98] Sharma P, Goswami S, Raychaudhuri D (2023). Immune checkpoint therapy—current perspectives and future directions. Cell.

[99] Yaghmour G, Pandey M, Ireland C (2016). Role of Genomic Instability in Immunotherapy with Checkpoint Inhibitors. Anticancer Res.

[100] Alouani E, Mercier M, Flecchia C (2023). Efficacy of immunotherapy in mismatch repair-deficient advanced colorectal cancer in routine clinical practice. An AGEO study ESMO Open.

[101] Jiang T, Zhou C, Ren S (2016). Role of IL-2 in cancer immunotherapy. Oncoimmunology.

[102] Madan RA, Gulley JL (2011). Sipuleucel-T: harbinger of a new age of therapeutics for prostate cancer. Expert Rev Vaccines.

[103] Kantoff PW, Higano CS, Shore ND (2010). Sipuleucel-T immunotherapy for castration-resistant prostate cancer. N Engl J Med.

[104] Burch PA, Breen JK, Buckner JC (2000). Priming tissue-specific cellular immunity in a phase I trial of autologous dendritic cells for prostate cancer. Clin Cancer Res.

[105] Brudno JN, Maus MV, Hinrichs CS (2024). CAR T Cells and T-Cell Therapies for Cancer: A Translational Science Review. Jama.

[106] Laetsch TW, Maude SL, Rives S (2023). Three-Year Update of Tisagenlecleucel in Pediatric and Young Adult Patients With Relapsed/Refractory Acute Lymphoblastic Leukemia in the ELIANA Trial. J Clin Oncol.

[107] Dreyling M, Fowler NH, Dickinson M (2024). Durable response after tisagenlecleucel in adults with relapsed/refractory follicular lymphoma: ELARA trial update. Blood.

[108] Munisvaradass R, Kumar S, Govindasamy C, Alnumair KS, Mok PL (2017). Human CD3_+_ T-Cells with the Anti-ERBB2 Chimeric Antigen Receptor Exhibit Efficient Targeting and Induce Apoptosis in ERBB2 Overexpressing Breast Cancer Cells. Int J Mol Sci.

[109] Wilkins A, Ost P, Sundahl N (2021). Is There a Benefit of Combining Immunotherapy and Radiotherapy in Bladder Cancer?. Clin Oncol (R Coll Radiol).

[110] Postow MA, Callahan MK, Barker CA (2012). Immunologic correlates of the abscopal effect in a patient with melanoma. N Engl J Med.

[111] Formenti SC, Rudqvist NP, Golden E (2018). Radiotherapy induces responses of lung cancer to CTLA-4 blockade. Nat Med.

[112] Zhang Z, Liu X, Chen D, Yu J (2022). Radiotherapy combined with immunotherapy: the dawn of cancer treatment. Signal Transduction and Targeted Therapy.

[113] Reits EA, Hodge JW, Herberts CA (2006). Radiation modulates the peptide repertoire, enhances MHC class I expression, and induces successful antitumor immunotherapy. Journal of Experimental Medicine.

[114] Sinha M, Zhang L, Subudhi S (2021). Pre-existing immune status associated with response to combination of sipuleucel-T and ipilimumab in patients with metastatic castration-resistant prostate cancer. J Immunother Cancer.

[115] Darragh LB, Knitz MM, Hu J (2022). A phase I/Ib trial and biological correlate analysis of neoadjuvant SBRT with single-dose durvalumab in HPV-unrelated locally advanced HNSCC. Nat Cancer.

[116] Spigel DR, Faivre-Finn C, Gray JE (2022). Five-Year Survival Outcomes From the PACIFIC Trial: Durvalumab After Chemoradiotherapy in Stage III Non-Small-Cell Lung Cancer. J Clin Oncol.

[117] Bradley JD, Nishio M, Okamoto I (2019). PACIFIC-2: Phase 3 study of concurrent durvalumab and platinum-based chemoradiotherapy in patients with unresectable, stage III NSCLC. Journal of Clinical Oncology.

[118] Yu Y, Zakeri K, Lee N (2021). Javelin Head Neck 100: Should we combine immunotherapy with radiation therapy?. Oncotarget.

[119] Lee NY, Ferris RL, Psyrri A (2021). Avelumab plus standard-of-care chemoradiotherapy versus chemoradiotherapy alone in patients with locally advanced squamous cell carcinoma of the head and neck: a randomised, double-blind, placebo-controlled, multicentre, phase 3 trial. Lancet Oncol.

[120] Telarovic I, Yong CSM, Kurz L (2024). Delayed tumor-draining lymph node irradiation preserves the efficacy of combined radiotherapy and immune checkpoint blockade in models of metastatic disease. Nature Communications.

[121] Shen Y, Connolly E, Aiello M (2025). Combination radiation and αPD-L1 enhance tumor control by stimulating CD8_+_ PD-1_+_ TCF-1_+_ T cells in the tumor-draining lymph node. Nature Communications.

[122] Chen J, Levy A, Tian AL (2025). Low-dose irradiation of the gut improves the efficacy of PD-L1 blockade in metastatic cancer patients. Cancer Cell.

